# Supplementary material for: Carvacrol-Induced Vacuole Dysfunction and Morphological Consequences in Nakaseomyces glabratus and Candida albicans
Source: Microorganisms. 2023 Dec 4;11(12):2915. doi: 10.3390/microorganisms11122915 (PMC10745442; doi:10.3390/microorganisms11122915)
Supplement: Supplementary file 1 [file microorganisms-11-02915-s001.zip › microorganisms-2714125-Supplementary.pdf]

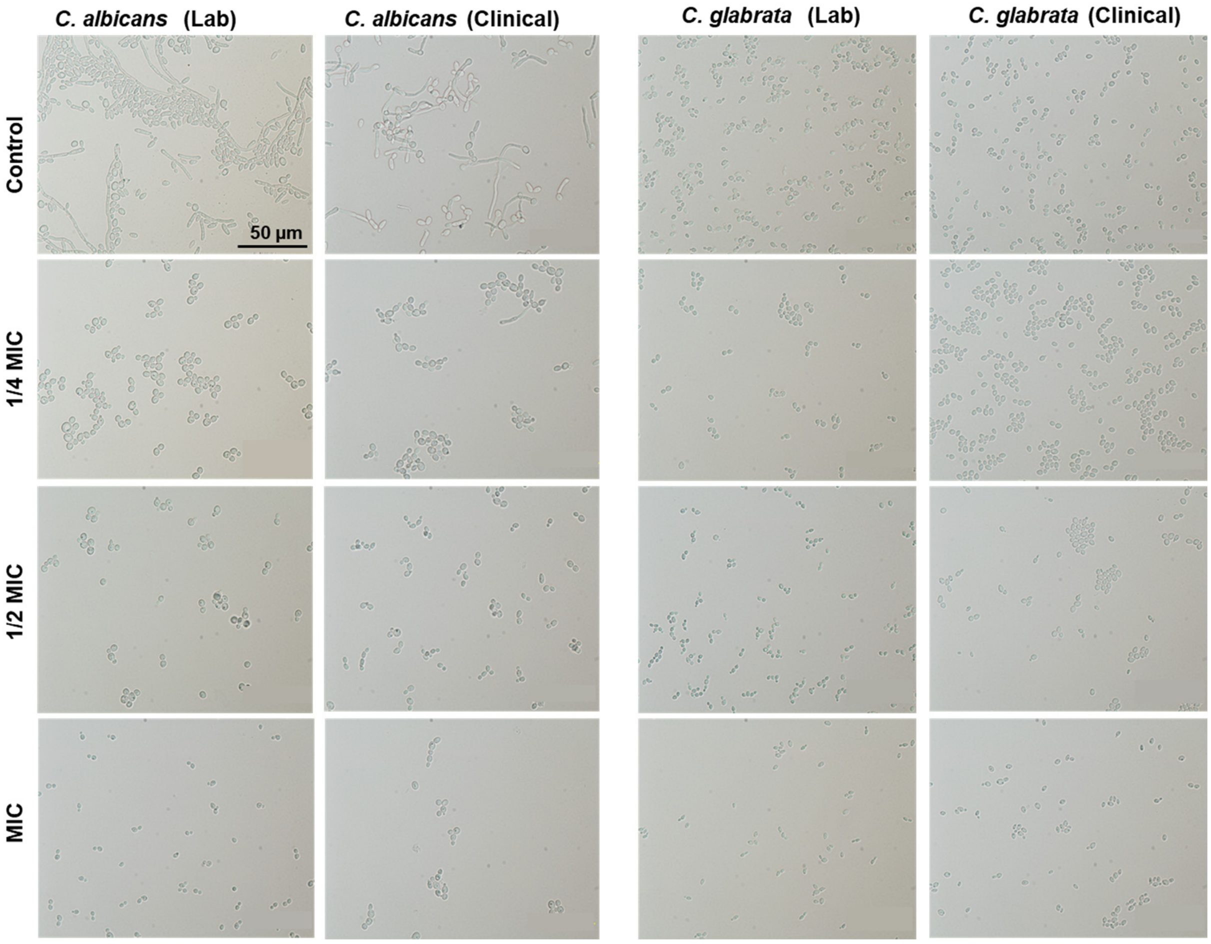

Figure S1. Effect of carvacrol on *C. albicans* and *N. glabratus* lab and clinical strains morphology. Bright-field images of control and carvacrol-treated cells following carvacrol treatment in YPD with 10% FBS. Scale bar is 50 µm.
